# Secondary Phase Precipitation in Fe-22Mn-9Al-0.6C Low-Density Steel during Continuous Cooling Process

**DOI:** 10.3390/ma17030631

**Published:** 2024-01-28

**Authors:** Yihao Zhou, Tinghui Man, Jun Wang, Hongshan Zhao, Han Dong

**Affiliations:** School of Materials Science and Engineering, Shanghai University, Shanghai 200444, China; zyh13657406066@163.com (Y.Z.); wj1415477560@shu.edu.cn (J.W.); boyushankf@126.com (H.Z.); 13910077790@163.com (H.D.)

**Keywords:** Fe-22Mn-9Al-0.6C low-density steel, secondary phase, precipitation, cooling rate, hardness

## Abstract

Secondary phase precipitation in Fe-22Mn-9Al-0.6C low-density steel was investigated during a continuous cooling process with different cooling rates through a DIL805A thermal expansion dilatometer, and the changes in microstructures and hardness by different cooling rates were discussed. The results showed that the matrix of the Fe-22Mn-9Al-0.6C was composed of austenite and δ-ferrite; moreover, the secondary phases included κ-carbide, β-Mn and DO_3_ at room temperature. The precipitation temperatures of 858 °C, 709 °C and 495 °C corresponded to the secondary phases B2, κ-carbide and β-Mn, respectively, which were obtained from the thermal expansion curve by the tangent method. When the cooling rate was slow, it had enough time to accommodate C-poor and Al-rich regions in the austenite due to amplitude modulation decomposition. Furthermore, the Al enrichment promoted δ-ferrite formation. Meanwhile, the subsequent formation of κ-carbide and β-Mn occurred through the continuous diffusion of C and Mn into austenite. In addition, the hardness of austenite was high at 0.03 °C/s due to the κ-carbide and β-Mn production and C enrichment, and it was inversely proportional to the cooling rate. It can be concluded that the presence of κ-carbide, DO_3_ and β-Mn produced at the austenitic/ferrite interface when the cooling rate was below 0.1 °C/s resulted in κ-carbide and β-Mn precipitating hardly at cooling rates exceeding 0.1 °C/s, which provides a guideline for the industrial production of Fe-Mn-Al-C low-density steel in the design of the hot working process.

## 1. Introduction

The pursuit of lightweight materials stands as a paramount concern in the design of vehicle structures and components, driven by environmental degradation and the diminishing reserves of fossil resources. While advancements in automotive steels have focused on augmenting strength, it has become evident that this alone is insufficient for achieving the desired lightweight properties. In recent decades, considerable attention has been directed towards low-density steels, distinguished by their exceptional combination of low mass and remarkable strength and ductility attributes. The addition of aluminum (Al) at a density of 2.7 g/cm^3^ induces lattice expansion within the steel matrix, resulting in an increased molar volume and a consequent reduction in density. Nonetheless, it is imperative to note that the solubility of Al in pure iron at ambient temperature is limited to approximately 9 wt.%. Consequently, efforts to enhance its solubility necessitate the incorporation of other elements with a high degree of austenite stability, such as manganese (Mn) [1,2,3,4,5,6,7,8,9]. Within the realm of materials, the Fe-Mn-Al-C series steel represents a noteworthy category, characterized by low density and exceptional strength [10]. This system predominantly fine-tunes alloy composition through the introduction of lightweight alloying elements, notably aluminum (Al) and silicon (Si) [11,12,13], yielding a novel automotive steel plate endowed with low density, high strength, and superior toughness. The overarching objective is the minimization of vehicular self-weight without compromising the structural integrity of essential components. Among these materials, austenitic dual-phase lightweight steel, celebrated for its heightened elongation characteristics, has garnered particular research focus due to its potential for revolutionary advancements in lightweighting strategies.

Austenitic dual-phase low-density steel is a noteworthy example, where the cooling rate plays a pivotal role in determining its properties. Liu et al. [14] demonstrated the ability to manipulate the size, volume fraction, and distribution of κ-carbide by varying the cooling rate, thus influencing deformation mechanisms and microstructure evolution. Ren et al. [15] employed two distinct cooling modes to control the precipitation of κ-carbides. They conducted a comprehensive study on the mechanical properties and deformation substructure evolution during tensile deformation in various states, elucidating the impact of early κ-carbide formation on both mechanical properties and deformation mechanisms. The findings indicate that the influence of cooling rate on the microstructure at the grain scale can be Park et al. [16] conducted a study using a Fe-30Mn-XAl-0.9C alloy with an aluminum (Al) content ranging from 9.0 to 12.8 wt%. The research focused on the impact of post-solution treatment cooling on the microstructure and mechanical properties of lightweight steel. The findings indicate that, with decreasing cooling rates, the alloy’s hardness increases due to the precipitation of κ-carbides. However, the reduction in cooling rate leads to the growth of intergranular κ-carbides. Consequently, the alloy demonstrates reduced impact absorption energy at room temperature, manifested as transgranular brittle fracture behavior. In Fe-Mn-Al-C low-density steel, carbide and ordered phases constitute significant precipitates that hold potential for enhancing the material’s overall mechanical properties [17,18,19,20]. Additionally, the deformation mechanism of low-density steel during tensile deformation is influenced by the shape, distribution, and volume fraction of precipitation [21,22,23,24,25,26,27,28].

**Figure 1:**
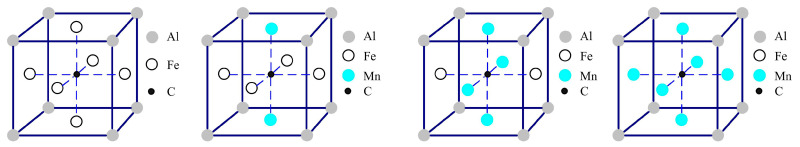
Lattice diagram of Fe_3-x_Mn_x_AlC (0 ≤ x ≤ 3)carbide in Fe-Mn-Al-C low-density steel.

**Figure 2:**
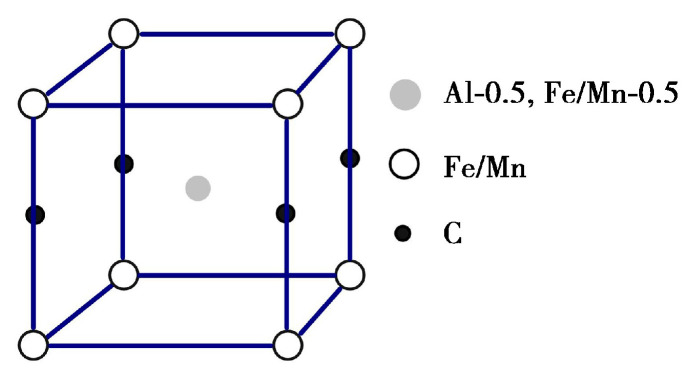
Lattice diagram of B2 ordered phase in Fe-Mn-Al-C low-density steel.

**Figure 3:**
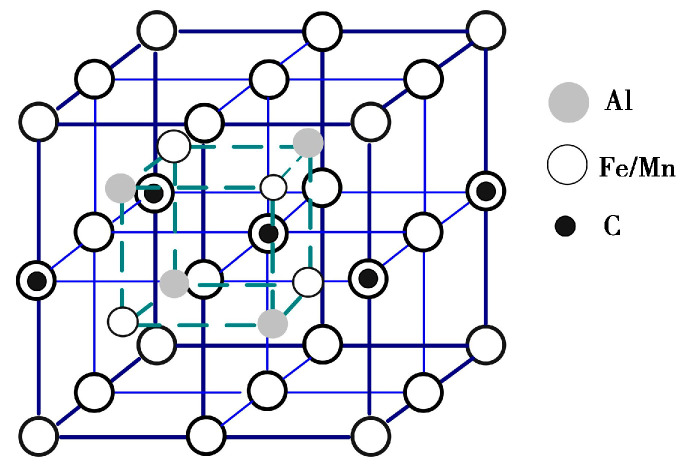
Lattice diagram of DO_3_ ordered phase in Fe-Mn-Al-C low-density steel.

Austenitic dual-phase low-density steel primarily derives its strength from solid solutions, fine grain structures, precipitation, and strain mechanisms. The precipitation strengthening is mainly attributed to the reinforcement effects of B2 and DO_3_ phases, along with κ-carbides. Among these, κ-carbides result from the presence of carbides such as M_3_C, M_5_C_2_, M_23_C_6_, and others in Fe-Mn-C steel. Upon the addition of Al to the steel, the Fe-Mn-Al-C alloy system is established. With an increase in Al content (>6.0%), the supersaturated austenitic steel, abundant in Al, Mn, and C, undergoes amplitude modulation decomposition during quenching or aging. The results are in the orderly arrangement of C and Al atoms, and the precipitated phase shifts from M_3_C-type carbide (Fe,Mn)_3_C to κ-carbides. κ-carbide is a perovskite cubic crystal structure of the E2_1_ type with a molecular formula of Fe_3_AlC. It is based on the L1_2_-type FCC-γ austenite, where the Al atom occupies the eight vertex corners of the cube, the Fe/Mn atom occupies six face center positions, and the C atom is located at the central position of the cubic crystal cell. This results in the formation of L′1_2_-type cubic crystal phases, such as Fe_3_AlC, Fe_2_MnAlC, FeMn_2_AlC, Mn_3_AlC, and others. The molecular formula is expressed as Fe_3-x_Mn_x_AlC (0 ≤ x ≤ 3), with a density ranging from 6.53 g/cm^3^ to 6.69 g/cm^3^, and the lattice structure is illustrated in Figure 1 [29]. Simultaneously, as the Al and C content changes, ferrite undergoes lattice ordering transformation, leading to the formation of the (Fe,Mn)_3_Al phase with a DO_3_ structure and the (Fe,Mn)Al phase with a B2 structure. The ordered phase B2 belongs to the MA_l_ type (M represents Fe, Mn, Ni, or (Fe,Mn) solid solution) intermetallic compound. Specifically, the FeAl phase within this category exhibits a density of 5.56 g/cm^3^, an elastic modulus of 259 GPa, a high specific modulus, and specific strength. It also possesses relatively low Gibbs free energy, resulting in a strong precipitation tendency. The plasticity of the B2 phase at room temperature is limited, but it excels in corrosion resistance, oxidation resistance, and wear resistance. Unlike κ-carbide, the B2 phase cannot undergo shear deformation, leading to dislocation accumulation at the phase interface. This, in turn, increases the processing hardening rate of low-density steel containing the B2 phase during deformation. The B2 phase adopts a bcc lattice structure, as illustrated in Figure 2 [30]. In the Fe-Al phase diagram, the presence of the Al element stabilizes α-Fe. With an atomic fraction of Al exceeding 20%, a transition occurs from a bcc disordered structure to ordering, resulting in the formation of a stable Fe_3_Al phase with a DO_3_ structure, possessing a density of 6.79 g/cm^3^. The hardness of the DO_3_ phase is relatively low (below 25 HRC), yet it exhibits a high work hardening rate and good wear resistance. The stability of this phase is temperature-dependent. Above 540 °C, the Fe_3_Al phase with the stable DO_3_ structure transforms into the FeAl phase with the B2 structure. Moreover, when the atomic fraction of Al surpasses 37%, this transformation also occurs into the FeAl ordered phase at room temperature. In the range of 23~37% atomic fraction of Al, coexisting precipitates of B2 and DO_3_ are observed. Below 540 °C during aging treatment, a slow transformation of B2→DO_3_ takes place. In Fe-Mn-Al-C low-density steel, Mn atoms dissolve within the Fe base, and the resulting precipitated phase with a DO_3_ structure is identified as (Fe,Mn)_3_Al, as illustrated in Figure 3 [31]. A study has demonstrated that κ-carbides at the nanoscale have no discernible impact on the plane slip mode [31]. As dislocations interact with ordered κ-carbides, additional dislocation sources begin to engage in cross-slip, promoting plane slip due to the uniform distribution of nanoscale shear κ-carbides that are coherent with the austenite matrix. The progressive refinement of the plane dislocation structure as strain increases constitutes the primary driver behind the continued strain hardening of κ-carbide-reinforced steel. Conversely, low-density steel containing B2 phase experiences rapid hardening during processing. This is attributed to the inability of B2 phases, unlike κ-carbides, to be shared by slip dislocations, resulting in the accumulation of dislocations at the phase boundary [28]. Yang et al. [32] proposed that the elevated work hardening rate in Fe-16Mn-10Al-0.86C-5Ni steel arises from the substantial back stress induced by the strain incompatibility between the austenite matrix and the second phase of B2 ordered metal.

The influence of continuous cooling on carbide and ordered phase precipitation, as well as the characteristics of low-density steel, have been relatively understudied. In their chemical analysis of Fe-0.77Mn-7.10Al-0.45C-0.31Nb, Gurgel, M.A.M. et al. [33] identified eutectoid ferrite (p), δ-ferrite, retained austenite, and niobium carbide (NbC) at various cooling rates (1 °C/s, 3 °C/s, 5 °C/s, 10 °C/s, 15 °C/s, 20 °C/s, and 50 °C/s). At lower cooling rates (1 °C/s and 3 °C/s), the combination of α-ferrite and κ-carbide revealed multilayer colonies of eutectoid microcomponents. Conversely, higher cooling rates (5 °C/s to 50 °C/s) resulted in the presence of martensite with body-centered cubic (BCC) and body-centered tetragonal (BCT) structures. In a continuous cooling thermal expansion experiment, Man et al. [34] investigated the influence of cooling rate on the precipitation behavior of κ-carbide in Fe-32Mn-11Al-0.9C low-density steel. It was observed that κ-carbide initiated precipitation at the γ/δ interface when the cooling rate decreased to 3 °C/s. Subsequently, both the quantity and size of κ-carbide exhibited significant increments, with further reductions in cooling rate. This led to a substantial increase in the nano-hardness of δ-ferrite due to the precipitation and hardening of κ-carbide at the γ/δ interface, while the nano-hardness of austenite experienced a marginal decline. In this study, we examined the effects of various cooling rates on the microstructure, properties, and precipitation of carbide and ordered phases in Fe-22Mn-9Al-0.6C low-density steel, aiming to achieve an optimal alignment of process parameters with mechanical characteristics.

## 2. Experiments

**Figure 4:**
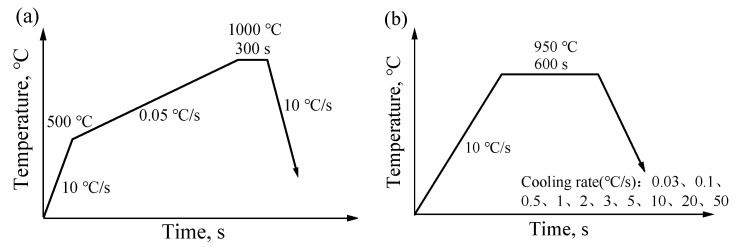
Schematic illustration of the continuous cooling process: (**a**) thermal expansion test; (**b**) continuous cooling curve.

The Fe-22Mn-9Al-0.6C low-density steel used in this study was melted in a 300 kg vacuum induction furnace and cast into a round bar of φ40 mm × 300 mm. The round bar was machined into a cylinder of φ4 mm × 10 mm to study the precipitation behavior during cooling using a DIL805A thermal expansion instrument. The experimental procedure involved some steps: first, the sample was heated to 500 °C at a rate of 10 °C/s; then, it was further heated from 500 °C to 1000 °C at a rate of 0.05 °C/s, and held at 1000 °C for five minutes before being cooled down to room temperature at a rate of 10 °C/s. Figure 4a is a schematic of the experimental procedure.

A set of ten samples was subjected to testing utilizing the DIL805A thermal expansion instrument for the assessment of Fe-22Mn-9Al-0.6C low-density steel. The thermal expansion curve during continuous cooling at various rates was also captured. The specimens underwent heating to 950 °C at a rate of 10 °C/s, followed by cooling to room temperature at diverse rates of 0.03 °C/s, 0.1 °C/s, 0.5 °C/s, 1 °C/s, 2 °C/s, 3 °C/s, 5 °C/s, 10 °C/s, 20 °C/s, and 50 °C/s, as depicted in Figure 4b. The tangent method was employed for the analysis and processing of the thermal expansion curve, enabling the determination of the temperature at which secondary phases precipitated. Post-testing, the samples underwent polishing and etching with an 8% nitric acid-alcohol solution for microstructural assessments. The microstructures and their alterations were scrutinized through the employment of LEICA DM2700M optical microscope (OM), THERMOFISHER Apreo 2S HiVac scanning electron microscope (SEM), and BRUKER Quantax 200 XFlash 6|60 Energy spectrometer (EDS). Subsequently, the WILSON VH1102 automatic microhardness tester was applied for hardness measurements of the samples’ matrix, employing a 0.5 kg load. More than ten points were evaluated for each sample, with the reported value representing the average. Ultimately, the phase compositions of distinct samples were assessed using a RIGAKU Smartlab X-ray diffractometer with a Cu target. The power was set at 9 kW, the testing angles ranged from 20° to 100°, and the scanning speed was 5°/min.

## 3. Results and Discussion

### 3.1. Hardness

**Figure 5:**
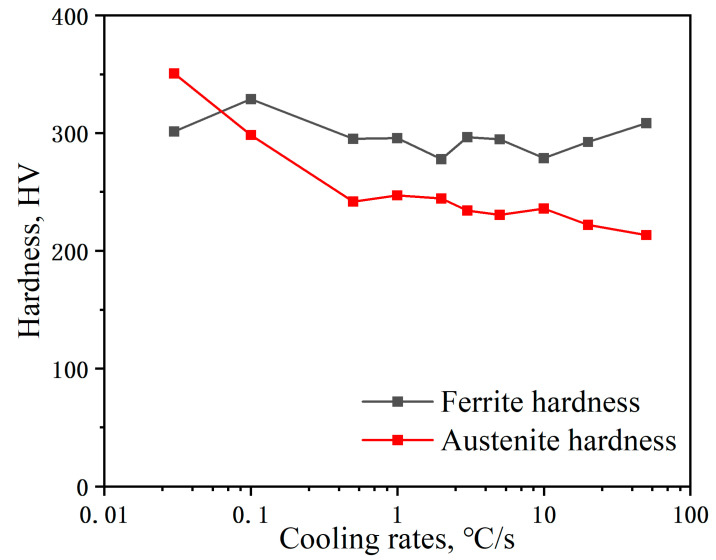
Hardness of Fe-22Mn-9Al-0.6C low-density steel with different cooling rates.

In Figure 5, the Vickers hardness variation curve is presented for both austenite and Fe-22Mn-9Al-0.6C low-density steel in relation to cooling rate. It can be observed from Figure 5 that the hardness of δ-ferrite in Fe-22Mn-9Al-0.6C low-density steel undergoes only a slight change, hovering around 300 HV, as the cooling rate spans from 0.03 to 50 °C/s. Additionally, Figure 5 illustrates a general decline in the austenite hardness of Fe-22Mn-9Al-0.6C low-density steel as the cooling rate escalates from 0.03 °C/s to 50 °C/s. This trend arises from the substantial precipitation of the β-Mn ordered phase and κ-carbide within the austenite matrix at lower cooling rates, which improves the hardness of the matrix. In contrast, at higher cooling rates, there is not enough time to precipitate the β-Mn ordered phase and κ-carbide, leading to a comparatively smaller amount in the matrix that cannot play a role in precipitation strengthening. Therefore, it results in a reduction in hardness.

### 3.2. Secondary Phase Precipitation

**Figure 6:**
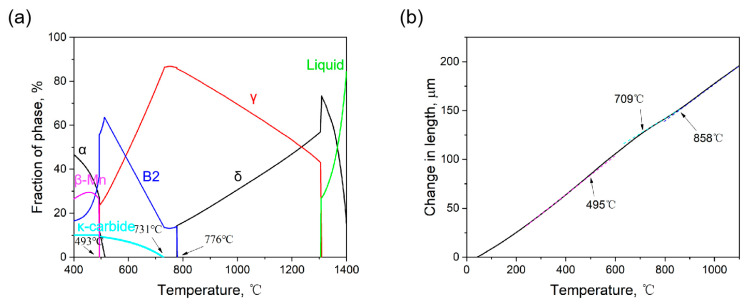
(**a**) Thermo-Calc equilibrium state property diagram; (**b**) Expansion curve.

The Thermo-Calc equilibrium state property diagram, illustrated in Figure 6a, delineates the precipitation temperatures of B2, κ-carbide, and β-Mn in Fe-22Mn-9Al-0.6C low-density steel at 776 °C, 731 °C, and 493 °C, respectively. Figure 6b displays the expansion curve of the steel, manifesting three distinct inflection points at 858 °C, 709 °C, and 495 °C.

### 3.3. Microstructural Characterization

**Figure 7:**
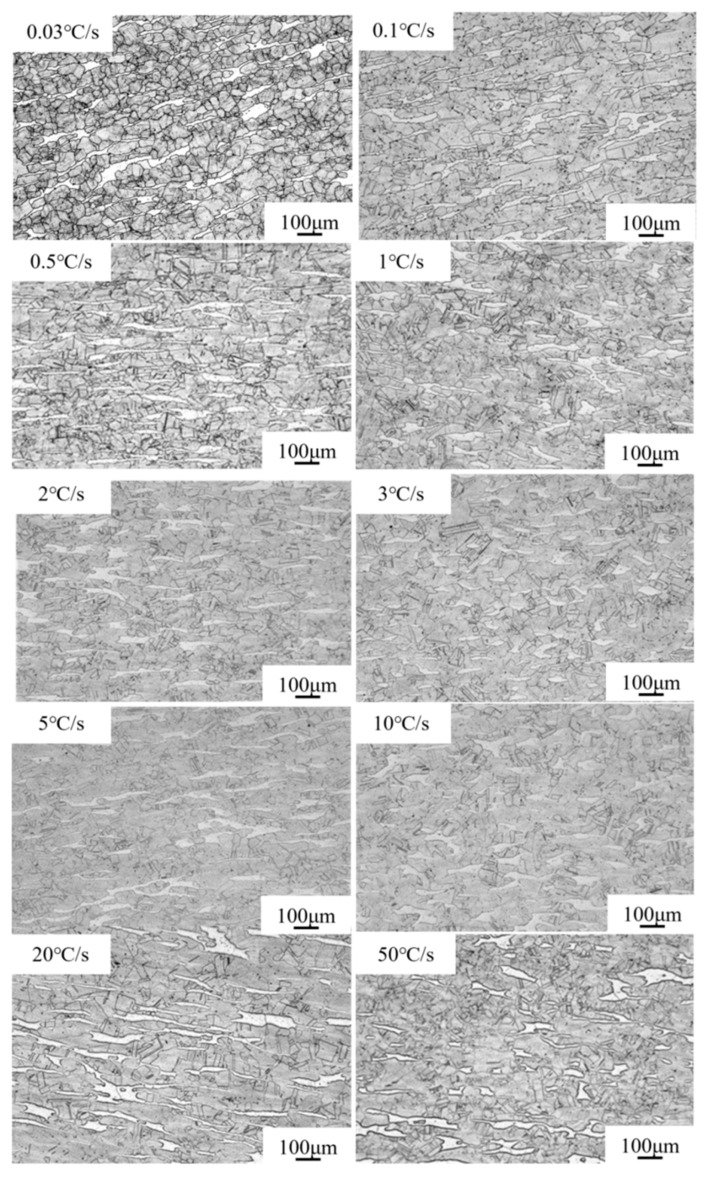
OM images of the microstructure under different cooling rates.

Fe-22Mn-9Al-0.6C low-density steel’s microstructure is depicted in Figure 7 at various cooling rates. It is found that δ-ferrite and austenite exist in the matrix for various cooling rates, and the size of the austenite grains increases with increasing cooling rates. The austenite is evenly equiaxed at a cooling rate less than 0.1 °C/s, which is due to the occurrence of sufficient recrystallization at 0.03 °C/s and 0.1 °C/s. The cooling rate was higher than 0.1 °C/s, the mixed crystal phenomenon occurred, subgrain boundaries emerged, and the banded δ-ferrite dispersed throughout the structure. In addition, the relatively fine austenite grains coexisted around the δ-ferrite, indicating that the surrounded recrystallized austenite grains were prevented by the banded δ-ferrite from growing.

**Figure 8:**
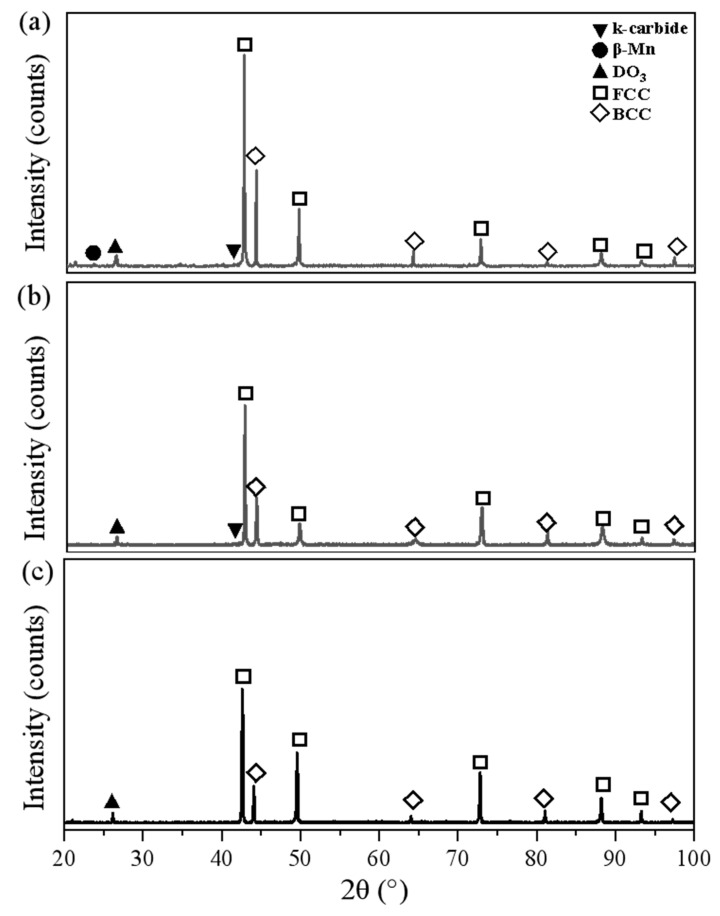
XRD diagram of Fe-22Mn-9Al-0.6C low-density steel at (**a**) 0.03 °C/s, (**b**) 0.1 °C/s, (**c**) 50 °C/s.

Figure 8a,b are XRD diagrams obtained for 0.03 °C/s and 0.1 °C/s that showed diffraction peaks corresponding to the (110), (200), (211), and (220) planes of a BCC structure at diffraction angles of 44.1°, 64.1°, 83.6°, and 97.4°, respectively, and diffraction peaks corresponding to the (111), (200), (311), and (222) planes of an FCC structure at diffraction angles of 42.6°, 49.6°, 72.7°, and 93.1°, respectively. In addition, they showed diffraction peaks at the degrees of 23.4°, 26.3°and 42.2°, corresponding to the DO_3_-ordered phase plane (111), the κ-carbide plane (111), and the β-Mn-ordered phase plane (111), respectively. In the investigated test conditions with continuously varying cooling rates (0.5 °C/s, 1 °C/s, 2 °C/s, 3 °C/s, 5 °C/s, 10 °C/s, 20 °C/s, and 50 °C/s), the diffractograms exhibited similar patterns to those shown in Figure 8c, which corresponds to a cooling rate of 50 °C/s. Notably, at intermediate and high cooling rates, the formation of κ-carbide and β-Mn was suppressed, as evidenced by the absence of peaks associated with these carbides. These peaks were, however, discernible at lower cooling rates of 0.03 and 0.1 °C/s. Specifically, the diffractograms revealed peaks corresponding to the (110), (200), (211), and (220) planes of a BCC structure at diffraction angles of 44.1°, 64.1°, 83.6°, and 97.4°, respectively. Additionally, they exhibited diffraction peaks corresponding to the (111), (200), (311), and (222) planes of an FCC structure at diffraction angles of 42.6°, 49.6°, 72.7°, and 93.1°, respectively. The reduction in austenite hardness in Fe-22Mn-9Al-0.6C low-density steel is attributed to the diminished precipitation of κ-carbide and β-Mn ordered phases as the cooling rate increases. This, in turn, results in a decline in the overall properties of Fe-22Mn-9Al-0.6C low-density steel.

**Figure 9:**
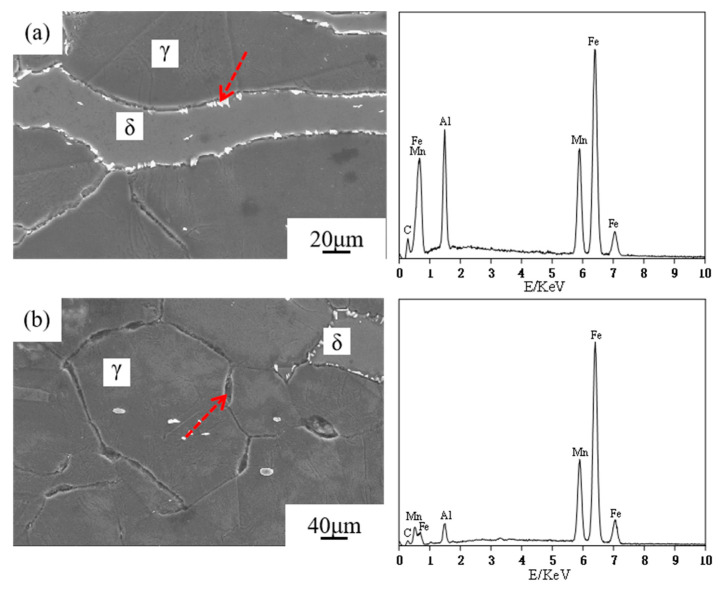
SEM images and corresponding energy spectrum of different precipitates of the steel at cooling rate of 0.03 °C/s: (**a**) DO_3,_ (**b**) κ-carbide.

Figure 9 depicts the secondary electronic SEM images and EDS analyses of the precipitates in the matrix. The EDS data demonstrate that there are two types of precipitates at the grain boundary: the dazzling white precipitated phase and the black precipitated phase. The bright white component has a high Al content, and the atomic ratio of Al:Fe is 1:3, indicating that the white particles are DO_3_-ordered. Because the atomic weight is low for Al and high for Fe and Mn, the black precipitates can be identified as an ordered phase of κ-carbide, which exists only at a cooling rate of 0.03 °C/s and 0.5 °C/s. As the cooling rate increases, grain boundary precipitation diminishes and turns into δ-ferrite intragranular precipitation.

**Figure 10:**
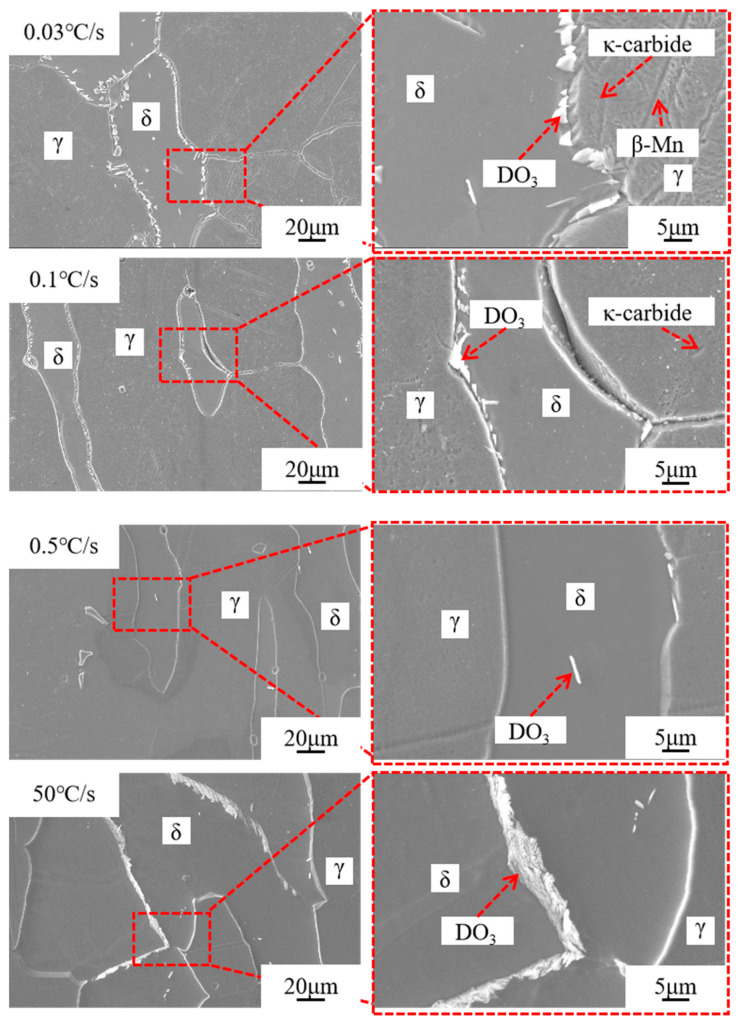
The microstructure of the steel at varying cooling rates.

Figure 10 shows the microstructure detected by SEM for samples with cooling speeds of 0.03 °C/s and 0.1 °C/s. The microstructure of these samples is typically composed of a greater relief matrix caused by austenite, as well as high relief regions (δ-ferrite) and precipitates. The number and size of precipitates grow as the cooling rate decreases, and the majority of the precipitates are mostly situated at the grain boundaries of δ-ferrite and austenite. At the same time, at a cooling rate of 0.03 °C/s, κ-carbide and β-Mn can be formed in the austenite matrix and at the grain boundary. The content of κ-carbide and β-Mn decreases significantly as the cooling rate increases. Figure 10 depicts the microstructure detected by SEM for samples with cooling rates of 0.5 °C/s and 50 °C/s. For these conditions, the relief (δ-ferrite) and higher relief (austenite), as well as the precipitates in δ-ferrite, can be inferred. The precipitated phase at the grain border of δ-ferrite and austenite disappears in the second stage and is replaced by the precipitated phase in the δ-ferrite crystal, and the size and quantity of the precipitated phase grow as the cooling rate increases.

It displays diffraction peaks indexed with both FCC and BCC structures for all cooling speeds in Figure 10. It is noteworthy that the diffraction peaks for samples subjected to low cooling rates (0.03 °C/s and 0.1 °C/s) differ from those with medium to high rates (0.5 °C/s to 50 °C/s). Specifically, carbide phases characteristic of the Fe-Mn-Al-C alloy were observed at low cooling rates but were absent at rates ranging from 5 to 50 °C/s. The diffraction peak of the FCC structure is attributed to the austenite phase, while δ-ferrite is a consistent component of the BCC structure at all cooling speeds.

Figure 10 exhibits a microstructure comprising a precipitated phase, a low-relief matrix, and a high-relief matrix. Based on observations and morphological traits in the literature, the matrix is presumed to be austenite. In the low-relief regions, it manifests as δ-ferrite. The occurrence of δ-ferrite at elevated temperatures and its persistence down to room temperature are well-documented phenomena. Additionally, the chemical composition of the alloy influences its stability. The elevated proportion of δ-ferrite is attributed to the high concentration of aluminum in the investigated alloy, a factor known to stabilize the δ-ferrite phase [24,35,36]. According to Jiang and Xie [37], both α-ferrite and δ-ferrite exhibit higher Al concentrations in the banded structure corresponding to δ-ferrite in the low-density steel alloy Fe-0.4C-1.5Mn-4Al (by weight).

Significant microstructural variations are evident in Fe-22Mn-9Al-0.6C lightweight steel under different cooling rates. Specifically, the austenite-to-ferrite ratio reaches its minimum at a cooling rate of approximately 0.1 °C/s. Subsequently, as the cooling rate decreases further to 0.03 °C/s, there is a subsequent increase in the ratio of austenite to ferrite, accompanied by the precipitation of κ-carbide and β-Mn from both the austenite and the phase boundary. The presence of κ-carbide in the austenite crystal contributes to the enhancement of austenite hardness. According to Chen [38], the initiation of δ-ferrite nucleation at austenite grain boundaries is facilitated by the breakdown of amplitude modulation in the austenite. This, in turn, encourages the development of numerous zones rich in C-poor and Al-rich phases. Subsequently, Al atoms migrate from the β-Mn phase to the ferrite, while C and Mn elements continue to diffuse from the β-Mn phase into the austenite, creating a Mn-rich area. Consequently, the distribution of C, Mn, and Al elements promotes Mn atomic segregation in the β-Mn phase, high Al content in δ-ferrite, and elevated C content in the austenite. Notably, C atoms undergo long-range diffusion in the austenite, while Al and Mn atoms exhibit short-range diffusion. At the junction of each β-Mn phase, a portion of Al atoms is released to generate new ferrites, initiating a new cycle. The β-Mn phases at various locations continuously amalgamate. Following an extended aging process, δ-ferrite and β-Mn mutually catalyze the formation of a substantial quantity of β-Mn. At a cooling rate of 0.03 °C/s, β-Mn has sufficient time for development. The heightened concentration of the C element in the precipitated austenite of β-Mn results in an increased hardness of the austenite.

## 4. Conclusions

As the cooling rate increases, the hardness of austenite in the Fe-22Mn-9Al-0.6C low-density steel decreases, while the hardness of δ-ferrite changes less. κ-carbide, DO_3,_ and β-Mn appear at the interface between austenite and ferrite when the cooling rate is below 0.1 °C/s. Conversely, κ-carbide and β-Mn have rarely precipitated as the cooling rate exceeds 0.1 °C/s. The precipitation of secondary phases in the Fe-22Mn-9Al-0.6C low-density steel can be controlled by adjusting the cooling rate, and the reinforcement from secondary phases contributes to improving the mechanical properties of the Fe-22Mn-9Al-0.6C low-density steel, providing a foundation for hot working during industrial production.

## Data Availability

The data presented in this study are available upon request from the corresponding author.
